# Complete chloroplast genome sequence and phylogenetic analysis of *Torenia fournieri*

**DOI:** 10.1080/23802359.2021.1939179

**Published:** 2021-06-14

**Authors:** Gang Chen, Li-guo Wang, Ying-hua Wang

**Affiliations:** a College of Life Sciences, Zhaoqing University, Zhaoqing, Guangdong, China; b Laboratory of Germplasm Resources and Molecular Identification of Traditional Chinese Medicine, School of Pharmaceutical Sciences, Guangzhou University of Chinese Medicine, Guangzhou, Guangdong, China

**Keywords:** Chloroplast genome, phylogenetic analysis, *Torenia fournieri*

## Abstract

*Torenia fournieri* belongs to the genus *Torenia* in the family Linderniaceae. The complete chloroplast genome of *T. fournieri* was sequenced and analyzed by Illumina sequencing in this study. The full length of the complete chloroplast genome is 153,938 bp, containing a pair of inverted repeat regions of 24,805 bp (IRa and IRb) separated by a large single copy region (LSC) of 85,498 bp and a small single copy region (SSC) of 18,830 bp. The *T. fournieri* chloroplast genome encodes 131 genes, comprising 87 protein-coding genes, 36 tRNA genes, 8 rRNA genes, without pseudogene. Phylogenetic analysis showed that *T. fournieri* was closely related to *T. benthamiana and T. concolor* within the genus *Torenia* in family Linderniaceae.

*Torenia fournieri* is one species of the genus *Torenia* (Linderniaceae), which distributes predominantly in Fujian, Guangdong, Guangxi, Taiwan, Yunan and Zhejiang provinces in China (Wu and Raven 1998). *Torenia fournieri* is not only an important tropical and subtropical ornamental plant, but also used as a model plant widely applied for flower research (Nishihara et al., 2018). *Torenia fournieri* used in this paper was planted in the Biological Garden of College of Life Sciences of Zhaoqing University (N23°6′, E112°30′, Zhaoqing, China), and the specimens (No: BGCLSZU001) were deposited in GDZQU herbarium of Zhaoqing University.

Firstly, the chloroplast genome DNA of *T. fournieri* was extracted from young leaves, and Covaris M220 (Covaris, Woburn, MA, USA) was used for breaking the DNA into about 300 bp fragments. Secondly, we constructed shotgun sequencing libraries according to the TruSeq™ DNA Sample Prep Kit for Illumina. Thirdly, whole genome sequencing was executed using the Illumina NovaSeq platform (Illumina, USA) (Genepioneer Biotechnologies Co. Ltd, Nanjing, China). Pair-end Illumina raw reads were cleaned from adaptors and barcodes and then quality filtered using Trimmomatic (Bolger et al. 2014). Then, reads were mapped to the chloroplast genome of the reference species (Genbank accession number: NC_045273.1), and the reads of nuclear and mitochondrial origins were excluded using Bowtie2 v2.2.4 (Langmead and Salzberg 2012). SPAdes 3.10.1 were used reconstruct the chloroplast genomes with *de novo* assembly method (Bankevich et al. 2012), and chloroplast contigs were concatenated into larger contigs using Sequencher 5.3.2 (Gene Codes Inc., Ann Arbor, MI, USA). A ‘genome walking’ technique, using the Unix ‘grep’ function, was used to find reads that could fill any gaps between contigs that did not assemble in the initial set of analyses (Souza et al. 2019). Jellyfish v.2.2.3 was used to correct misassembled contigs (Marcais and Kingsford 2011). Annotation of the chloroplast genomes were generated by CpGAVAS (Liu et al. 2012) and a circular representation was drawn with the online tool OGDRAW (Lohse et al. 2007). The complete chloroplast genome sequence has been submitted to Genbank with the accession number of MW307826.

The length of chloroplast genome sequence of *T. fournieri* is 153,938 bp, including two inverted repeat regions (IRa and IRb, each 24,805 bp) separated by a LSC (85,498 bp) region and a SSC (18,830 bp) region. The GC content of the overall chloroplast genome, IR regions, LSC, and SSC are 37.57, 43.45, 35.39 and 32.00%, respectively. The chloroplast genome contains 131 genes in total, including 87 protein-coding genes, 36 tRNAs, 8 rRNAs, without pseudogene.

The 15 whole genomes were used for phylogenetic tree analysis. First, we used MAFFT v7.427 (Katoh et al. 2005) -auto mode to align each sequence. The gaps in the alignment were removed using the program trimAl with ‘-nogaps’ v 1.4 (Capella-Gutierrez et al. 2009). Finally, Maximum-likelihood (ML) method was used to construct the phylogenetic tree with MEGA v7.0 (Kumar et al. 2016); the nucleotide substitution model used was the Kimura 2-parameter model with 1000 bootstrap replicates (Figure 1). We found that *T. fournieri* was closely related to *T. benthamiana and T. concolor* within the genus *Torenia* in family Linderniaceae.

**Figure 1.**
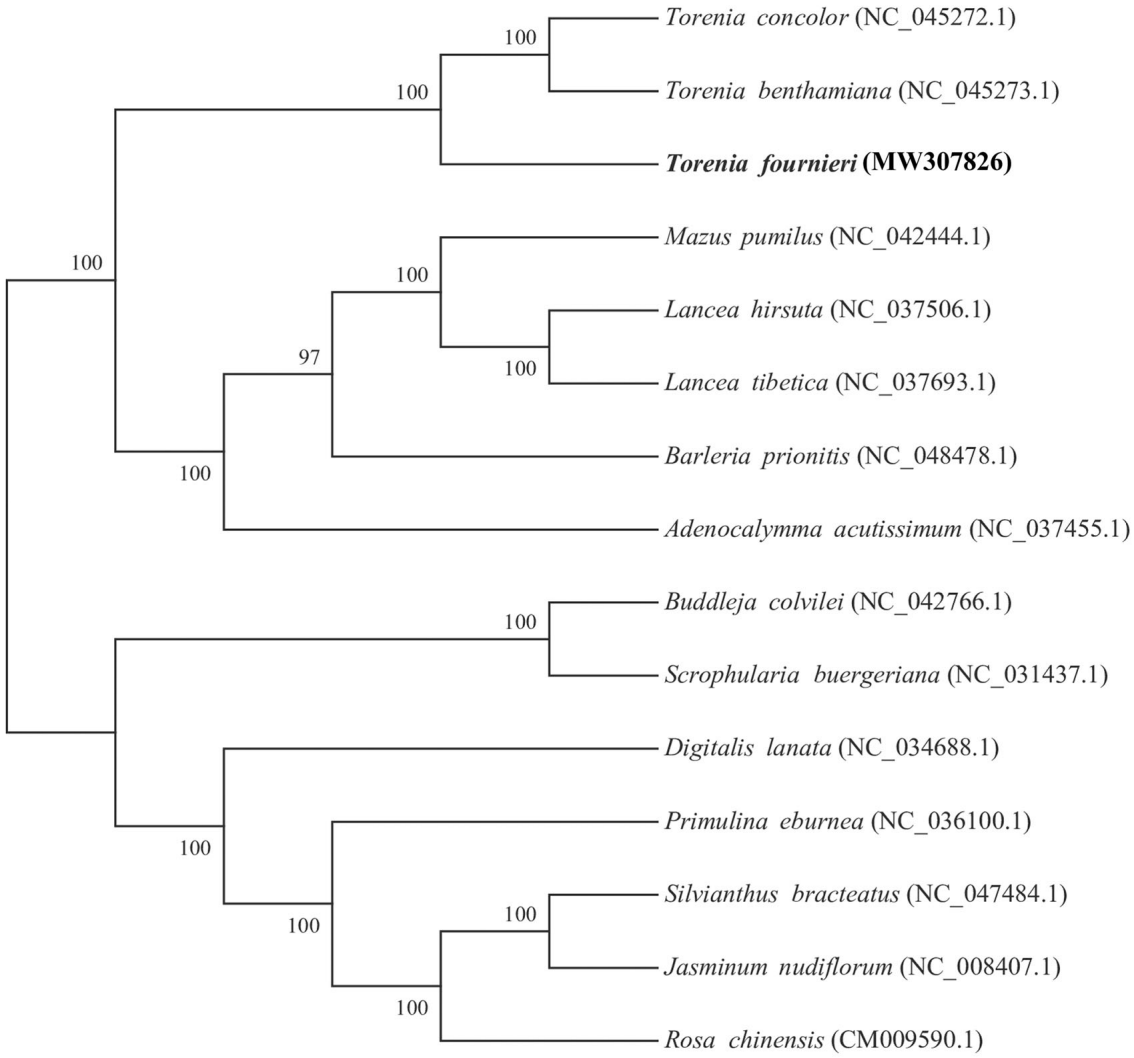
Phylogenetic tree reconstruction of 15 species based on sequences from whole chloroplast genomes. All the sequences were downloaded from NCBI Genbank.

## Data Availability

The data that newly obtained at this study are available in the NCBI under accession number of MW307826 (https://www.ncbi.nlm.nih.gov/nuccore/MW307826).
